# Effect of Information Framing on Wearing Masks During the COVID-19 Pandemic: Interaction With Social Norms and Information Credibility

**DOI:** 10.3389/fpubh.2022.811792

**Published:** 2022-02-23

**Authors:** Lihong Peng, Hao Jiang, Yi Guo, Dehua Hu

**Affiliations:** Department of Biomedical Informatics, School of Life Sciences, Central South University, Changsha, China

**Keywords:** COVID-19, information framing, framing effect, mask wearing, information credibility, social norms

## Abstract

**Objective:**

The main objectives of this study were to use the effect of information framing (different expressions of the same issue, e.g., positive messages and negative messages) to explore key factors that influence the attitude of and intention of the public toward wearing masks and to understand the internal and external factors of intervention on information framing perception.

**Methods:**

This study performed an online questionnaire survey to explore the influence of demographic characteristics, information framing, social norms, and information credibility on the attitude of the public toward masks and their intention to wear them.

**Results:**

(1) Information framing had a significant impact on the attitudes of people toward masks and their intention to wear them, and the persuasion effect of gain-framed messages was higher than that of loss-framed messages. (2) Gender, income, occupation, educational background, and residence have no significant difference in attitude and intention to wear masks. There was a significant correlation between age and wearing of masks (*p* = 0.041 < 0.05). (3) Social norms affected people's perception of information framing and their attitude toward wearing masks, but only the impact of loss-framed messages on intention was significant. (4) Information framing affected people's perception of information credibility, which had a positive impact on their intention to wear masks; however, information credibility only had a significant impact on attitude toward wearing masks under the gain-framed messages and played an intermediary role.

**Conclusion:**

The impact of information framing on the attitude of people toward masks and their intention to wear them varies. Individuals involved in the publicity of health information related to this issue should pay attention to the influence of information framing and content on the public wearing masks as a means of enhancing public health awareness.

## Introduction

As of September 1, 2021, there were 218.59 million confirmed cases of coronavirus disease-2019 (COVID-19), and 4.53 million deaths were caused by the disease. The virus spreads in various ways. Apart from common droplets and contact transmission, aerosol transmission may occur in confined spaces, which poses challenges to our ability to prevent and control the pandemic. Wearing masks is an effective self-protection behavior in the fight against infectious diseases, and it is listed as an important protective measure. The use of masks is also valuable in the control of infectious diseases, especially with respect to avoiding the spread of droplets (1, 2). At present, the literature on wearing masks is mostly focused on determinants of wearing masks and seek to improve the use of masks in order to establish effective intervention measures that help block the epidemic or similar airborne infectious diseases (3).

While focusing on the “Asian Disease Problems”, Kahneman and Tversky first put forward the concept of “information framing”, which asserts that individual risk preference often depends on the expression of the problem (4). “Gain-loss framing”, which emphasizes the benefits of accepting certain health behaviors (gain framing), and losses incurred by rejecting certain health behaviors (loss framing), has become the most widely used type of health behavior information framing. A study asserted that loss framing is more effective in promoting disease detection behaviors, and that gain framing is the best method to use to encourage disease-prevention behaviors (5). This view has aroused widespread debates among scholars, and many argumentation studies on this topic have emerged. Through six experiments, a research study proved that when information is devoted to promoting certain behaviors, gain framing is more convincing, and that when information is used to prevent a certain phenomenon, loss framing is more effective (6). The presentation of information framing offers a new theoretical perspective for information behavior research as well as a new research concept that can be used while exploring the influence of information on behavior-related decisions.

Throughout the COVID-19 pandemic, research has approached the connection between wearing masks and information framing through political, cultural, and ethical lenses. Exploring cultural framing involves assessing if cultural factors affect mask-wearing behaviors (7). Martinelli (8) studied the social cultural, ethical, and political components of wearing masks by investigating how these factors influence public health policies and determining the best ways to account for these factors in health information dissemination. A research study on societal values and mask usage in the context of COVID-19 control in the United States found that mask-wearing is divisive and politicized (9). However, other researchers have demonstrated that when people place a high degree of trust in their governments, which is the case in South Korea and China (10, 11), preventive actions are rarely politicized. Because our investigation was conducted in China, we did not emphasize political factors in our assessment. Steffen (12) studied the influence of information framing on wearing masks during the COVID-19 period and considered the potential influence of political ideology, computing ability of a supervisor, and risk attitude. However, these studies are only based on hypothetical ideas (such as how many people not wearing masks will die and how many lives can be saved by wearing masks) rather than actual experiences. A research study on social pressure, altruism, free-riding, and noncompliance with mask-wearing was conducted, and the results show that promotion of altruism is more likely to increase mask-wearing than social shaming (9). However, no study has discussed how the credibility of a specific content affects the decision-making behavior of people. One of the aims of this study is, thus, to explore the influence of the content credibility of information framing on behavior decision-making. Because of the COVID-19 pandemic, the preventive behavior of people may also be influenced by policies and social relationships. Consequently, the second goal of this study is to consider how social norms affect people's perception of information framing and impact their attitude and intention toward wearing masks.

In summary, this study investigated the health behaviors the public during the COVID-19 pandemic by analyzing mask-wearing behaviors. In doing so, we reveal the internal and external factors of intervention on information framing perception and identify differences in cognition and decision-making caused by the influence that information framing has on the decision-making process of wearing masks. Developing a deeper understanding of how information framing affects the process of health behavior decision-making during the pandemic and determining the role that it plays in the construction of health behavior attitudes and intentions not only improve the research theory of information framing and health behavior but also offer a scientific basis for health behavior intervention programs.

## Materials and Methods

### Investigation Method

Two different questionnaires (loss framing [version A] and gain framing [version B]) were distributed through the online questionnaire platform WJX. All research team members participated in the data collection process, and survey links were distributed through social software such as WeChat and QQ. In addition, participants were also required to share the questionnaire. The participants randomly received a questionnaire, which instructed them to not fill in volume B if they had already completed volume A, so as to eliminate the possibility of cross-filling. The questionnaire was distributed for 2 weeks, from September 3, 2021 to September 17, 2021.

All subjects read the informed consent form before filling out the questionnaire. If they agreed to answer the questions and submit the questionnaire online, it meant that they gave their informed consent. The study obtained ethical approval from the Institutional Review Board of the College of Life Sciences at Central South University (Reference No. 2020-1-44) and followed the guidelines of the Declaration of Helsinki. In order to be included in the study, participants needed to have a certain level of reading comprehension and offer their informed consent and voluntary participation. Individuals who suffered from mental illness or cognitive impairment or who refused to participate in the study were excluded. Before the survey took place, a pilot study was conducted offline on a community of 30 subjects to ensure that participants would not have difficulty reading the framed messages or understanding and answering the questions in the questionnaire. Most participants indicated that information framing was easy to understand, and that the length of the questionnaire was appropriate.

In the end, 445 valid questionnaires were obtained after removing invalid questionnaires. In this study, the Cronbach α coefficient and combination reliability (CR) in Smart PLS 3.0 were used to judge the reliability of this research model, and average variance extracted (AVE) was selected to test convergence validity. The value of Cronbach α coefficient and the combination reliability (CR) of all variables in this study are above 0.7, and the AVE values of all the variables are above 0.5. The above indicates that the reliability of the model scale is good and has good convergence validity, therefore, formal analysis can be conducted.

### Information Framing and Wearing Masks

All information involved in this study was designed according to the characteristics of the gain and loss frames based on literature review, resident interviews, and consultations with experts. In addition, to avoid the impact of different amounts of information, the number of words between the two frames was similar and controlled to about 120. Stimulation information is shown in Table 1.

**Table 1:** Stimulation information used in the questionnaire.

**Information**	**Gain-framed messages**	**Loss-framed messages**
1	The novel coronavirus is mainly transmitted through the respiratory tract, and the mask can play a preventive role, protecting ourselves and others.	The novel coronavirus is mainly transmitted through the respiratory tract. Without wearing a mask, you can't play a preventive role, which not only brings infection risk to yourself but also to others.
2	Wearing a protective mask in public places can block the spray nucleus containing the virus, which prevents the wearer from inhaling and thus reduces the probability of infection.	Without wearing a protective mask in public places, it is impossible to block the spray nucleus containing the virus, which means that you are likely to be invaded by the virus and infected with COVID-19.
3	If there is a virus carrier, wearing a mask can block the transmission route of the virus and prevent mass infection among gathered people.	If there is a virus carrier present, it is impossible to block the transmission route of the virus without wearing a mask, which means that not wearing one will cause mass infection among the gathered people.

### Model Building

**Figure 1:**
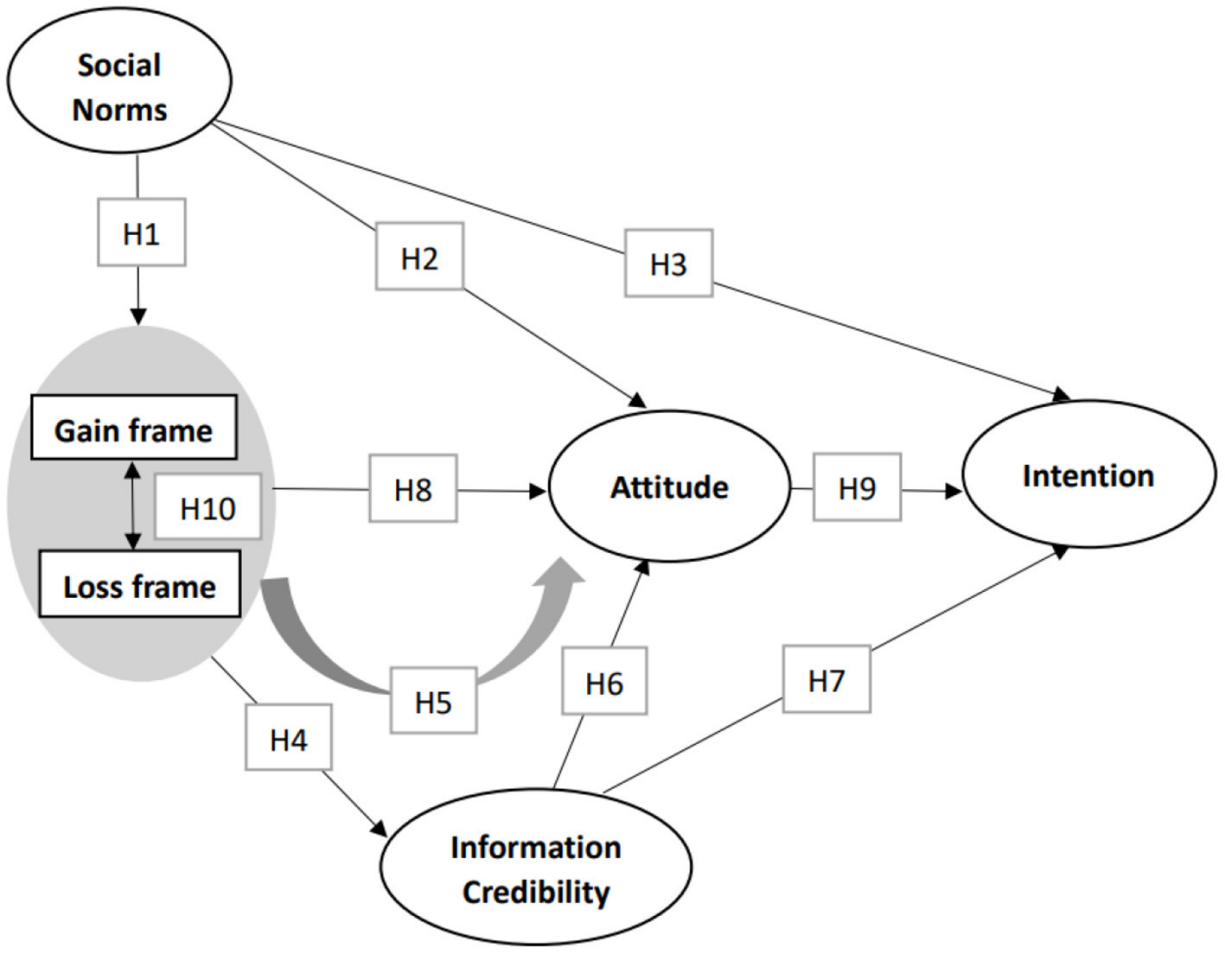
Structural equation model of this study.

In order to explore the impact of information framing effect on public health behavior during the COVID-19 pandemic, this study considered the external moderating role of social norms and the mediating role of information credibility while constructing a theoretical model (Figure 1). The research includes four types of variables. The independent variable is information framing (IF), the dependent variable is attitude (AT) toward wearing masks and intention (IT), and the intermediary variable is information credibility (IC). Social Norms (SNs) are also included in the model as an external influencing factor. We studied the above variables and put forward relevant assumptions.

### Research Hypothesis

#### Social Norms

Cialdini and Trost suggest that social norms are usually defined as codes of conduct that differ from laws and regulations and are generally accepted by group members (13). Social norms are rules or standards of behaviors that guide the actions of people and help promote social harmony by constructing expectations on how individuals should act (14). The influence of social norms on health behavior has attracted a significant amount of attention (15–17) and has been studied using different theoretical models, such as planned behavior theory (18) and normative social behavior theory (19). In the context of the COVID-19 pandemic, behavior is related to social norms (20), which are influenced by social values and choices that lead to change in views of people, including opinions of family members, relatives, and friends (21). Nabi found that the cognition of social norms affects the acceptance of information by an individual, and that people tend to accept information that is consistent with their social norms (22). Voisin et al. showed that when the information contained in intervention measures aligns with some social norms, it can effectively promote healthy behaviors (23). On the basis of previous studies, the following assumptions are put forward from the perspective of social norms:

H1: Social norms will affect the perception of information framing by participants.
H2: Social norms will affect the attitudes of the participants toward wearing masks.
H3: Social norms will affect the intention of the participants to wear masks.

#### Information Credibility

Information credibility is the judgment of an individual on the authenticity of information content, and the perception of information credibility by people further influences judgments that they make (24). When people receive information, they judge its credibility in different ways. Appelman proposed that individuals evaluate the reliability of information content according to its accuracy, authenticity, and credibility (24). Etingen found that there is a relationship between information credibility and behavior (25). Previous studies have also shown that there is a significant correlation between perceived credibility and self-protection behavior (26). A study on environmental protection found that highly credible gain-framed messages can actively shape the attitudes and intentions of people (27). When information appears to have low credibility, the intention of individual behavior decreases (28). The previous studies mentioned above have shown that information credibility does play an intermediary role in the behavior decisions of people. Therefore, this study puts forward the following assumptions:

H4: Information framing will affect the judgment of the participants on the credibility of information.
H5: Information credibility plays an intermediary role between information framing and wearing masks.
H6: Information credibility has a positive impact on the attitude of an individual toward wearing masks.
H7: Information credibility has a positive impact on the intention of an individual to wear masks.

#### Information Framing, Attitude, and Intention

Researchers usually design corresponding information framing according to different research purposes, and “gain-loss framing” has become the most widely used type of health behavior information framing. According to the research conducted by Tversky, the effect of framing proves that information expressed in the form of gain or loss has different influences on behavior decisions. Health behavior decision-making differs from risk decision-making in that it does not involve examining different alternatives that pose varying levels of risk; instead, individuals choose to accept or reject certain healthy (or unhealthy) behaviors, and “risk” manifests as subjective feelings about possible adverse consequences. Therefore, “gain-loss framing,” which emphasizes the benefits of accepting certain health behaviors (gain framing) and the losses caused by rejecting certain health behaviors (loss framing), has become the most widely used type of health behavior information framing (4).

According to the theory of planned behavior, attitude and intention can measure behavior to a certain extent (18). A study on information framing and consumers showed that if information framing describes the benefits of buying organic food or emphasizes the losses caused by not buying that type of product, then it will significantly affect the attitude of consumers and intention to purchase (29). In research that focuses on the relationship between corporate social responsibility problem and positive influence, problem participation is positively correlated with positive influence and is, therefore, positively correlated with attitude and behavior intention (30). In the field of health behavior, it has been found that intentions of college students to get vaccinated against HPV differ depending on the type of information that they receive (31). In a study on preventing skin cancer among teenagers, information framing influenced the intentions of subjects to wear sunscreen and trousers to prevent skin cancer (32). Therefore, information framing has a certain impact on attitudes of people toward particular behaviors and, thus, affects changes of intention. Van't Riet assessed if customization of intervention information can promote physical activity and if using gain or loss framing to publicize personal privacy produces different effects on information acceptance, attitude, intention, and behavior. The results showed that compared with loss framing, gain framing leads to stronger physical activity intention (33). Therefore, this study puts forward the following assumptions:

H8: Information framing will influence attitudes of the participants toward wearing masks.
H9: Attitudes of the participants toward wearing masks have a positive impact on their intention.

Rothman asserted that loss framing is more convincing in the context of disease detection behavior, and that gain framing is more convincing in situations related to disease prevention (5). This view has sparked a widespread debate among scholars, and many argumentation studies have been conducted on this topic. Through six experiments, Lee proved that when information is devoted to promoting certain behaviors, gain framing is more convincing, and that when information is used to prevent a certain phenomenon, loss framing is more effective (34). During the COVID-19 pandemic, a study showed that when it comes to encouraging the adoption of preventive behaviors, positive information framing usually promotes the desired behavior more efficiently (35). Wearing masks essential to prevent the spread of COVID-19. Therefore, this study puts forward the following assumptions:

H10: The persuasion effect of gain framing on wearing masks is higher than that of loss framing.

### Variable Measurement

This study focuses on information framing, attitude, and intention related to wearing masks, influence of social norms, and measurement of information credibility. All these factors were assessed using the Likert 5 scale; the higher the value, the higher the effect of information framing, the more positive the attitude and intention of wearing masks, the greater the influence of social norms and the higher information credibility perceived. Measurements for related factors were all adapted from existing research. Specifically, the measurement for information framing was adapted from the study of Gantiva et al. (36), including three measurement items, such as “I believe that not wearing a mask will increase my risk of COVID-19 infection” in the loss frame and “I believe that wearing a mask will reduce my risk of COVID-19 infection” in the gain frame. The measurement for assessing attitude toward adopting healthy behaviors was adapted from the research of Mir et al. (37), including three items: “In my opinion, wearing a mask is an effective measure to prevent the COVID-19 pandemic,” “I will not resist wearing a mask”, and “I think wearing a mask is a necessary behavior”. The measurement for intention to wear masks was adapted from the research of Steffen et al. (12) and included four items that appear in their study: “Based on the above statement about taking preventive measures, I decided to wear masks in public places,” “I would recommend my family and friends to wear masks,” “If the doctor/government suggests, I will strictly abide by the measures of wearing masks”, and “I intend to wear masks because it can block the spread of viruses”. The measurement for social norms was adapted from the research of Mir et al. (37) and included four items that appear in their study: “My family suggested that I should wear a mask,” “My friends and colleagues influenced my decision to wear a mask,” “Important people influenced my decision to wear a mask”, and “The policy of epidemic prevention and control guidelines influenced my decision to wear a mask.” The measurement for information credibility was adapted from the research of Appelman (24) and Sundar (38) and mainly measures the accuracy, authenticity, and reliability of information through items such as “I think the above information is”. In accordance with existing research, this study has six control variables: gender, age, income, occupation, residence, and education level.

## Results

### Influence of Demographic Characteristics on Wearing Masks

We collected 445 valid questionnaires. The effects of two variables (gender and residence) on wearing masks were tested by independent sample *t*-test, and the effects of four variables (age, income, occupation, and educational background) on wearing masks were tested by variance (ANOVA).

According to the results shown in Table 2, the compliance of women with wearing masks is higher than that of men (*M* = 4.32 > *M* = 4.21), and the compliance of urban residents with wearing masks is higher than that of rural residents (*M* = 4.29 > *M* = 4.22). With respect to income, people with highest income have lowest compliance. In addition, the higher the education level of an individual, the lower their compliance with wearing masks. In terms of occupation, the compliance of farmers with wearing masks is lowest. It is worth noting that there is a significant correlation between age and how compliant an individual is with wearing masks (*p* = 0.041 < 0.05). The older a person is, the more likely they are to be compliant with wearing masks.

**Table 2:** Influence of demographic characteristics on wearing masks.

**Variable**		***N* (%)**	***M* (SD)**	***t*/*F***	***p***
Gender	Man	141 (31.7%)	4.21 (1.210)	1.003	0.317
	Woman	304 (68.3%)	4.32 (1.062)		
Residence	City	378 (84.9%)	4.29 (1.112)	0.224	0.636
	Rural	67 (15.1%)	4.22 (1.112)		
Age	≤ 18	4 (0.9%)	3.75 (1.893)	2.521	0.041
	18–29	372 (83.6%)	4.23 (1.139)		
	30–49	50 (11.2%)	4.52 (0.931)		
	50–59	18 (4.1%)	4.89 (0.323)		
	≥60	1 (0.2%)	5.00 (0.000)		
Revenue (Yuan)	≤ 3,000	217 (48.8%)	4.32 (1.043)	0.641	0.634
	3,001–5,000	92 (20.7%)	4.22 (1.221)		
	5,001–10,000	82 (18.4%)	4.33 (1.078)		
	10,001–20,000	44 (9.9%)	4.27 (1.169)		
	>20,000	10 (2.2%)	3.80 (1.549)		
Occupation	Student	200 (44.9%)	4.25 (1.088)	1.791	0.113
	Civil servant	16 (3.6%)	4.50 (0.816)		
	Employees of enterprises/ institutions	137 (30.8%)	4.19 (1.179)		
	Self-employed/ Freelancer	24 (5.4%)	4.25 (1.327)		
	Farmers	10 (2.3%)	3.90 (1.595)		
	Others	58 (13%)	4.64 (0.831)		
Education	Junior college and below	57 (12.8%)	4.49 (1.120)	1.362	0.257
	Undergraduate	250 (56.2%)	4.28 (1.127)		
	Master's degree and above	138 (31%)	4.20 (1.075)		

### Model Hypothesis Testing

**Figure 2:**
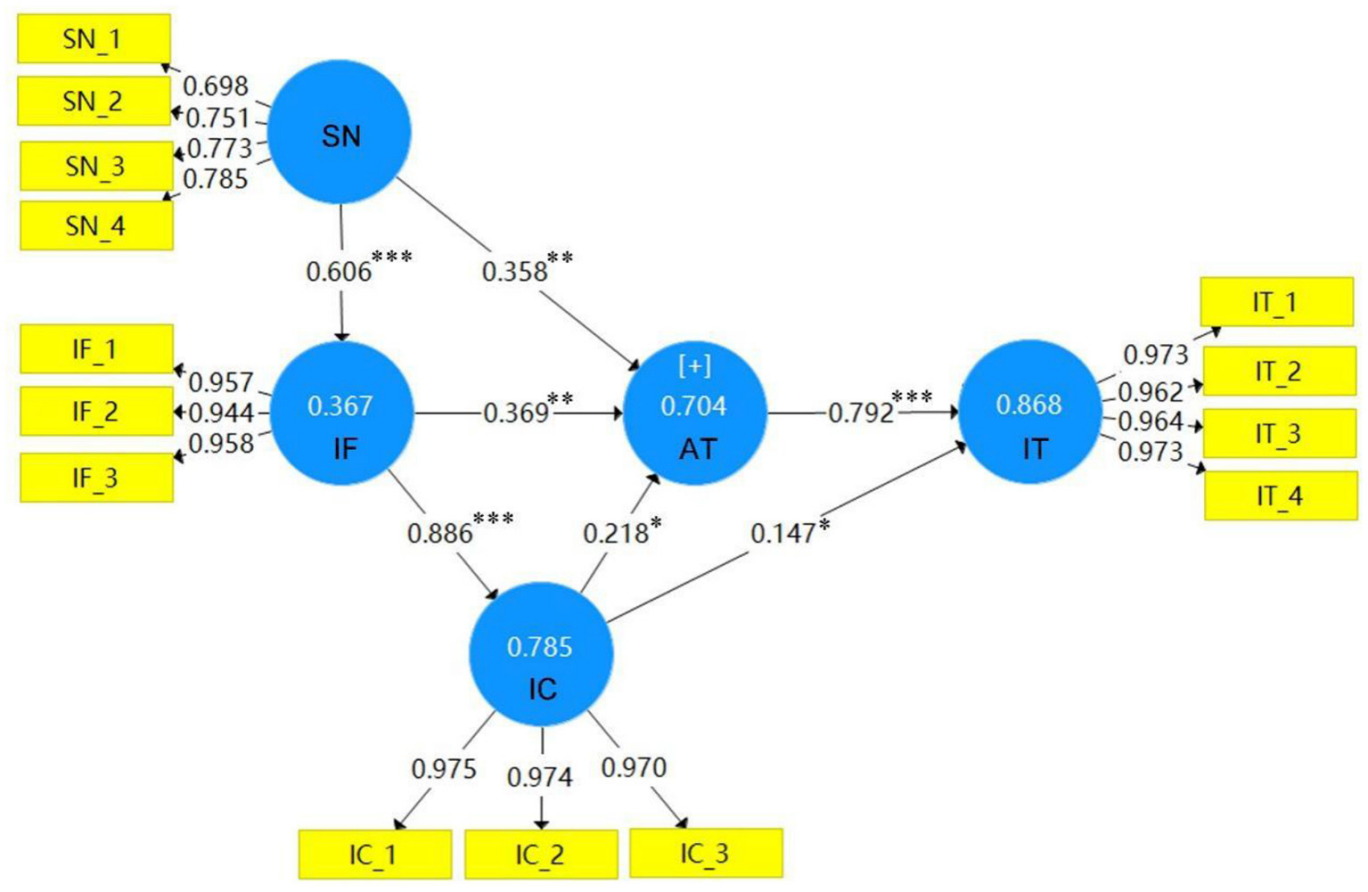
Research model of hypothesis testing of gain framing. *p < 0.05, **p < 0.01, ***p < 0.001.

**Figure 3:**
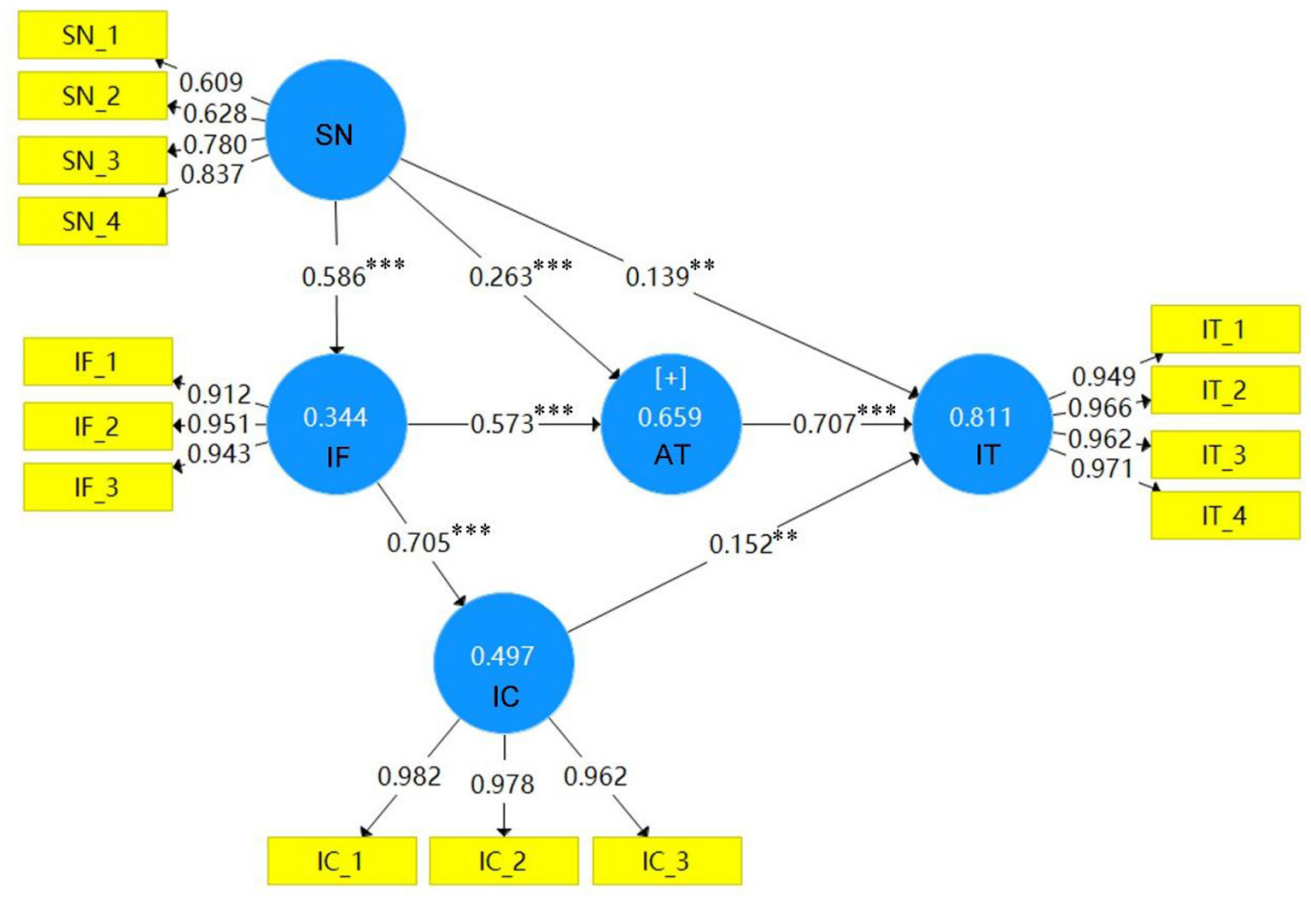
Research model of hypothesis testing of loss framing. *p < 0.05, **p < 0.01, ***p < 0.001.

In this study, Smart PLS 3.0 was used to test whether each path hypothesis in the research model is valid, and the results are shown below in Figures 2, 3. The path still existing among variables indicates that the hypothesis on the path was established. Otherwise, the hypothesis was not established, so the path was removed from the research model. As shown in Figures 2, 3, the variables of this research model (information framing [IF], information credibility [IC], attitude [AT], and intention [IT]), and their explained variances are 36.7, 78.5, 70.4, and 86.8%, respectively, under gain framing; the explained variances under loss framing are 34.4, 49.7, 65.9, and 81.1%, respectively.

In order to confirm the hypotheses of the model, this study performed bootstrapping in Smart PLS 3.0 to observe numerical changes in each path, and *t*-test to judge the hypothetical results. If the calculated *t*-value is larger than 1.96, it means that the hypothesis in this path is valid. The support for each hypothesis is shown below in Table 3.

**Table 3:** Support of model path hypothesis.

**Hypothesis**	**Path**	**Information framing**	***t***	***p***	**Hypothetical content**	**Is the hypothesis established?**
**H1**	SN → IF	Gain	7.315	^***^	H1: Social norms will affect participants' perception of information framing	Yes
		Loss	11.571	^***^		Yes
**H2**	SN → AT	Gain	3.110	^**^	H2: Social norms will affect participants' attitudes toward wearing masks	Yes
		Loss	3.599	^***^		Yes
**H3**	SN → IT	Gain	1.034	0.301	H3: Social norms will affect participants' intention to wear masks	No
		Loss	2.839	^**^		Yes
**H4**	IF → IC	Gain	24.222	^***^	H4: Information framing will affect the participants' judgment about the credibility of information	Yes
		Loss	8.479	^***^		Yes
**H6**	IC → AT	Gain	2.230	^*^	H6: Information credibility has a positive impact onan individual's attitudes toward wearing masks	Yes
		Loss	0.997	0.319		No
**H7**	IC → IT	Gain	2.212	^*^	H7: Information credibility has a positive impact on an individual's intention to wear masks	Yes
		Loss	3.276	^**^		Yes
**H8**	IF → AT	Gain	3.220	^**^	H8: Information framing will influence participants' attitudes toward wearing masks	Yes
		Loss	5.607	^***^		Yes
**H9**	AT → IT	Gain	11.571	^***^	H9: Participants' attitudes toward wearing masks have a positive impact on their intention	Yes
		Loss	9.907	^***^		Yes

* *p < 0.05,*

** *p < 0.01,*

*** *p < 0.001*.

#### Hypothesis Testing of Social Norms

With gain framing, social norms affected the participants' perception of information framing and their attitudes toward wearing masks. As shown in Figures 2, 3, with β values of 0.606 and 0.358, *t*-values of 7.315 and 3.11, H1 and H2 were both established. Social norms had no significant influence on intention to wear masks, with β = 0.033 and *t*-value < 1.96, so H3 was not verified. As shown in Figure 3 and Table 3, social norms under loss framing affected the participants' perception of information framing and their attitude toward masks and intention to wear them, with β values of 0.586, 0.263, and 0.139, respectively, and *t*-values of 11.571, 3.599, and 2.839, respectively, H1, H2, and H3 were all established.

#### Hypothesis Testing of Information Credibility

Gain framing affected the participants' perception of the credibility of information and had a positive impact on their attitude and intention to wear masks. As shown in Figure 2 and Table 3, the β values are 0.886, 0.218, and 0.147, respectively, and the *t*-values are 24.222, 2.23, and 2.212, respectively, so the assumptions laid out in H4, H6, and H7 were all established. As shown in Figure 3 and Table 3, loss framing had a positive impact on information credibility and intention to wear masks, with β values of 0.705 and 0.152 and *t*-values of 8.479 and 3.276, respectively, meaning that the assumptions of H4 and H7 were established. Information credibility has no significant impact on the attitudes of participants toward wearing masks, with β = 0.077 and *t*-value < 1.96, so H6 was not verified.

#### Hypothesis Testing of Information Framing

As shown in Figures 2, 3, the β value of attitude toward wearing masks within gain and loss framing are 0.369 and 0.573, respectively. As shown in Table 3, the *t*-values are 3.22 and 5.607, respectively, so H8 was established. The attitudes of participants toward wearing masks has a positive impact on their intention, with β values of 0.792 and 0.707, and *t*-values of 11.571 and 9.907, respectively; therefore, assumption H9 was established.

### Test of Information Credibility Intermediary Effect

To evaluate the effect of mediation, we used variance accounted for (VAF). The meaning of VAF value is the proportion of indirect effect to overall effect (direct effect plus indirect effect). When the VAF value is <20%, it means that there is no median effect, while 20% < VAF value <80% indicates that there is a partial mediation effect, and VAF value>80% indicates that there is a complete mediation effect.

In this study, the VAF value under gain framing is 34.3%, which indicates that information credibility plays a partial mediating role in attitude, so H5 was established. Under loss framing, the indirect effect path (IF → IC → AT) is not significant (*p* = 0.367 > 0.05), and the VAF value is 8.8%, which indicates that information credibility has no mediating effect on attitude; thus, H5 was not verified. The results of the mediation effect test are shown below in Table 4.

**Table 4:** Intermediary effect test table.

**Independent variable**	**Mediator variable**	**Dependent variable**	**Direct effect**	**Indirect effect**	**Overall effect**	**VAF**	**Hypothesis**
Gain	Information reliability (IC)	Attitude (AT)	0.369^**^ (3.220)	0.193^*^ (2.262)	0.562^***^ (4.413)	34.3%	H5 established
Loss			0.573^***^ (5.607)	0.055 (0.902)	0.628^***^ (7.166)	8.8%	Not significant

* *p < 0.05,*

** *p < 0.01,*

*** *p < 0.001*.

### Persuasion Effect of Information Framing

The information framing involved in this study is divided into the gain-frame and loss-frame groups. As shown in Table 5, the attitude (*M* = 4.593, SD = 0.0) and intention (*M* = 4.679, SD = 0.719) of the gain-frame group are slightly lower than the attitude (*M* = 4.628, SD = 0.659) and intention (*M* = 4.685, SD = 0.685) of the loss-frame group. Information framing was significantly correlated with the attitude of the participants and their intention to wear masks (*p* < 0.001). However, the comparison between the mean values of these two framing groups is not obvious.

**Table 5:** Results of linear regression analysis.

**Variable**	**Information framing**	***M* (SD)**	**Adj.*R*^2^**	***B***	***t***	***p***
Attitude	Gain	4.593 (0.720)	0.605	0.779	18.385	<0.001
	Loss	4.628 (0.659)	0.605	0.779	18.513	<0.001
Intention	Gain	4.679 (0.719)	0.628	0.793	19.278	<0.001
	Loss	4.685 (0.666)	0.608	0.781	18.609	<0.001

Through further assessment by linear regression analysis, we found that with respect to attitude toward wearing masks, the absolute value of the standardized beta coefficient of the gain-frame group is equal to that of the loss-frame group (*B* = 0.779, *p* < 0.001). With respect to intention to wear masks, the value of the gain-frame group is larger than that of the loss-frame group (*B* = 0.793 > *B* = 0.781, *p* < 0.001). Overall, these results show that the gain-framed message is more effective on persuading people to wear masks, which means that the assumption laid out in H10 was established.

## Discussion

### Influence of Demographic Characteristics on Wearing Masks

The compliance of women with wearing masks is higher than that of men. The research of Bir research shows that in many situations, such as visiting grocery stores and schools, the proportion of women wearing masks is higher (9). With respect to these gender differences, people believe that women are usually less willing to take risks, so they are more willing to take preventive actions than men (39). A sizable amount of literature has been produced during the COVID-19 pandemic and previous epidemics that focus on compliance with social distancing, hygiene, and quarantine rules, and these studies show that men often have a lower rate of compliance than women (40, 41). However, in the case of receiving COVID-19 vaccination, men are more likely to get vaccinated than women, which may be associated with the fact that men have a higher risk of COVID-19 complications and death (42). In our opinion, another reason for this difference is that wearing a mask is a low-risk protective behavior, but receiving a vaccination carries high risks. If avoiding COVID-19 infection involves taking risks (e.g., possibly incurring side effects from a vaccination), the proportion of female compliance will be lower. Therefore, the reasons underlying the inconsistent results of gender compliance with preventive measures in many studies may be due to a range of factors, such as self-efficacy and risk preference, which are worthy of further exploration.

As for place of residence, urban residents are more compliant with wearing masks than rural residents, which is consistent with the findings of Ferdous et al. (43, 44), and residents living in urban areas also have a more positive attitude toward preventive measures. In terms of income, people with highest level of income have lowest degree of compliance. Those who are seriously worried about the virus (possibly because of potential medical conditions) are more likely to wear masks (12), but people with high incomes have sufficient funds to obtain better medical care, which means that their compliance is lower. Moreover, we found that individuals with higher level of education were less likely to be compliant with wearing masks. This research result goes against findings of previous research (45–47). The possible reason for this might be that most highly educated people (undergraduate and above) in this research sample are students in school. On the basis of closed management in Chinese universities, most students are only active in campus, and the behavior of wearing masks inside campus is less rigid. In terms of occupation, the compliance of farmers with wearing masks is lowest, which might be related to the nature of their job. There is a significant correlation with age (*p* = 0.041 < 0.05). Older individuals were more likely to comply with wearing masks. This may be because the risks of hospitalization, serious illness, and death caused by COVID-19 increase with age, and the elderly have the highest risk.

In summary, we should improve the compliance with wearing masks among male groups, rural residents, and farmers. We should also adopt preventative measures that encourage younger groups, high-income groups, and highly educated groups to wear masks. The willingness of an individual to wear masks may depend on certain contexts and levels of exposure. For instance, the low compliance of farmers and rural residents may stem from the fact that they are less exposed to people; however, it is still important to remind these types of groups to wear masks when they go out.

### Impact of Social Norms

Social norms affected people's perception of information framing and their attitude toward wearing masks under both frames of loss and gain. The assumption posed in H3 had inconsistent verification results under different frames. The reason for this may be that under loss framing, people's negative perception of loss information more strongly influenced their intention than the positive perception of equivalent gain information (48). Syed observed that peer pressure significantly affected the use of masks (49). After the government and public health officials promoted the use of masks, the frequency of mask-wearing in communities increased, especially during the outbreak of the disease (50–52). An explanation for this is that individuals are more tightly bound and tend to prioritize the needs of larger communities in countries that are more collectivistic (53). With respect to the influence of intention, loss framing had a significant effect on wearing a mask. This result is consistent with the study of Burgess et al. (50). Some Japanese people wear masks because they feel pressure from their families, doctors, and schools. When not wearing a mask has adverse effects on individuals (i.e., this behavior affects personal income/employment, etc.), their compliance with wearing masks will improve. When persuading people to wear masks through social relationships or public policies, it is more effective to use negative information (emphasizing the disadvantages of not wearing masks).

### Information Credibility and Intermediary Effect

The results show that information framing will lead people to judge the credibility of information and has a positive impact on their intention to wear masks, but the credibility of information has no significant impact on attitudes toward wearing masks under loss framing. The reason for the inconsistency of the H6 test results may be that, regardless of the credibility of information, loss framing heightened the perceived risk of individuals and made them more likely to wear masks.

Under gain framing, information credibility partially mediates attitudes toward wearing masks, but under loss framing, there is no significant correlation between information credibility and attitudes toward wearing masks, which leads to no intermediary effect. Therefore, when spreading persuasion-related information on wearing a mask, if we start with benefits of wearing a mask, we should pay attention to the accuracy and reliability of the information used, which will affect the effect of health communication.

On the whole, gain-loss framing triggers people's perception of information credibility, which has a positive impact on their intention to wear masks. However, only gain framing has a significant positive correlation with attitude of wearing masks. Similarly, under gain framing, the mediating effect of information credibility is valid. When persuading people to wear masks with positive information, people consider the credibility of information more carefully; therefore, we should pay attention to improving the accuracy, reliability, and authenticity of information. For example, speeches of public authorities and government departments can be incorporated into health communication strategies in order to enhance people's perception of the credibility of information and improve their attitude and intention to wear masks.

### Impact of Information Framing

The results of this study show that information framing affects the attitudes of people toward masks and their intention to wear them. The persuasion effect of wearing masks in the gain framing group was larger than that in the loss framing group. This result verifies previous studies that demonstrate that positive information has a larger persuasion effect than negative information when it comes to prevention behaviors (36). A study on social pressure, altruism, free-riding, and noncompliance in the context of mask wearing showed that individuals who believe wearing masks protect others were more likely to report that they voluntarily wore them, and perceiving social pressure negatively impacted the probability of voluntary mask-wearing (9). When the message emphasizes the benefits of wearing masks to oneself and others, the persuasion effect will be stronger than emphasizing the disadvantages of not wearing masks. Thus, to encourage people to wear masks, it is more effective to add gainmessages in health information dissemination.

Generally, information framing has an impact on health behaviors, and gain framing is more effective in promoting health prevention behaviors. Moreover, social norms can regulate the attitudes of people toward healthy behaviors, but they may not affect the intention of people to take self-protection measures. The mediating effect of information credibility plays a partial mediating role or does not play a mediating role in different behaviors and frames. The results of this study provide theoretical and practical references for promoting healthy preventive behaviors.

### Limitations and Prospects

This study describes the role of information framing in health communication and emphasizes the primary psychological and demographic factors of health behavior decision-making as well as the role that social norms and information credibility have played during the COVID-19 pandemic. However, this research has some shortcomings. In terms of research objects, most of the participants were young and middle-aged. The limitations of the sample may affect the popularity of the research results, so it is necessary to further explore the influence of information framing on the attitudes and intention of adolescents and the elderly to take preventive measures. In terms of research content, this study focuses on how information framing affects the mask-wearing behaviors of people, but in future research, we will compare a range of different types of behaviors, such as wearing masks, getting vaccinated, and maintaining social distance, in order to study the relationship and potential mechanism among these behaviors.

## Data Availability Statement

The raw data supporting the conclusions of this article will be made available by the authors, without undue reservation.

## Ethics Statement

The studies involving human participants were reviewed and approved by Institutional Review Board of College of Life Sciences, Central South University (Reference No.2020-1-44). Written informed consent to participate in this study was provided by the participants' legal guardian/next of kin.

## Author Contributions

LP and DH designed the study. HJ and LP led the investigation and formal analysis, and wrote the first version of the manuscript. YG and DH reviewed and edited the manuscript. DH was responsible for the administration of the project. All authors have read and agreed to the published version of the manuscript.

## Funding

This research was funded by the National Social Science Fund of China (Grant No: 20BTQ081), Key International Cooperation Projects of Hunan Province of China (Grant No: 2021WK2003), and the Project of Theory, Practice and Popularization of Scientific and Technological Novelty Search by Shenzhen Health Development Research and Data Management Center (Grant No: H202111120250001).

## Conflict of Interest

The authors declare that the research was conducted in the absence of any commercial or financial relationships that could be construed as a potential conflict of interest.

## Publisher's Note

All claims expressed in this article are solely those of the authors and do not necessarily represent those of their affiliated organizations, or those of the publisher, the editors and the reviewers. Any product that may be evaluated in this article, or claim that may be made by its manufacturer, is not guaranteed or endorsed by the publisher.
